# Gastric cancer survival prediction using artificial intelligence models based on electronic health records: a systematic review and meta-analysis

**DOI:** 10.3389/fdgth.2026.1845932

**Published:** 2026-06-23

**Authors:** Maryana Mandrina, Tigran Gevorkyan, Sergey Zvezda, Valeria Pavlova, Rukiyat Abdulaeva, Mariam Manukyan, Yana Belenkaya, Sergey Gordeyev, Ivan Stilidi

**Affiliations:** 1 N.N. Blokhin National Medical Research Center of Oncology, Moscow, Russia; 2 Tyumen State Medical University, Tyumen, Russia; 3 Sechenov University, Moscow, Russia

**Keywords:** artificial intelligence, electronic health records, gastric cancer, meta-analysis, survival prediction, systematic review

## Abstract

**Importance:**

Artificial intelligence (AI) is increasingly being applied to prognostic modeling in oncology; however, many AI-based survival prediction models rely on complex multimodal data that are not routinely available in clinical practice.

**Objective:**

This systematic review and meta-analysis aimed to evaluate the performance of AI models based on routinely collected electronic health record (EHR) data for predicting 5-year overall survival (5-OS) in patients undergoing surgical treatment for gastric cancer

**Data sources:**

A systematic literature search was conducted in PubMed, Scopus, Nature, MedRxiv, and bioRxiv databases for studies published between January 2015 and July 2025.

**Study selection:**

We included studies reporting area under the receiver operating characteristic curve (AUC) values for AI-based 5-OS prediction. Retrospective studies of adult patients with histologically confirmed gastric cancer who underwent curative-intent surgery were eligible, while studies primarily using non-routine multimodal data or lacking AUC outcomes were excluded.

**Data extraction and synthesis:**

Risk of bias was assessed using the PROBAST-AI tool. Meta-analyses were performed to compare machine learning-based models with conventional statistical approaches, as well as different AI algorithm classes, including bagging and boosting ensemble methods, neural networks, random forest, support vector machines, and logistic regression. Random or fixed-effects models were applied according to between-study heterogeneity.

**Main outcome(s) and measure(s):**

The primary outcome was the pooled mean difference in AUC between machine learning-based and conventional statistical models for 5-OS prediction. Secondary outcomes included comparative performance across different AI algorithm classes and identification of the most frequently selected prognostic features.

**Results:**

Ten retrospective studies comprising 15,643 patients were included. Machine learning-based models demonstrated a modest but statistically significant improvement in predictive performance compared with conventional approaches, with a pooled mean AUC increase of 0.04 (95% CI 0.02–0.07; *p* = 0.001). Boosting algorithms showed a modest but statistically significant advantage over bagging methods (AUC increase 0.02; *p* = 0.04). The type of clinical input data, particularly the inclusion of blood-based biomarkers, influenced algorithm performance. The most consistently identified prognostic features across studies were age, T stage, tumor size, serum albumin or prealbumin level, and metastatic-to-examined lymph node ratio.

**Conclusions:**

and relevance: AI-based prognostic models utilizing routinely available clinical data provide clinically meaningful improvements in 5-year survival prediction after gastric cancer surgery, and selection of the optimal AI algorithm should be guided by the structure and type of input data to maximize both predictive performance and practical applicability in clinical decision support systems.

**Systematic Review Registration:**

https://www.crd.york.ac.uk/PROSPERO/view/CRD420261282797, PROSPERO CRD420261282797.

## Introduction

Survival outcomes following primary treatment for localized and locally advanced gastric cancer remain modest. Currently, there is no universal model for predicting overall survival, and the widely accepted TNM staging system lacks sufficient predictive power to guide individualized treatment escalation or de-escalation for patients (1). With the advancement of artificial intelligence (AI) approaches, there is growing interest in its application for predicting oncological treatment outcomes. Particularly valuable is the use of readily available data from electronic health records (EHRs) for prognostication, which can be obtained without the need for additional processing.

However, the data from EHRs may be diverse and require different algorithms for optimal processing. Future research should prioritize identifying data features with the highest predictive value for AI-based prognostic models.

A recent meta-analysis examined the overall potential and limitations of AI-based gastric cancer (GC) prognostic models, comparing performance metrics and algorithms across various datatypes (2). While it revealed a high potential of the new technologies, a more focused approach is necessary to guide future research directions using different data types. Comparing performance outcomes across studies with different experimental setups, datasets and evaluation protocols misleading conclusions.

The present systematic review and meta-analysis aimed to: (a) evaluate the prognostic performance of AI models using EHR data for predicting 5-year overall survival (OS) in GC patients; (b) determine whether the type of input data influences the performance of different AI algorithms; and (c) compare predictive accuracy between boosting and bagging ensemble methods in AI-based prognostic models.

## Methods

The systematic review was conducted in accordance with the Preferred Reporting Items for Systematic Reviews and Meta-Analyses (PRISMA) 2020 guidelines (3). Ethical committee approval was not required for this study, as no patient identifiers were disclosed. The protocol was registered on PROSPERO (CRD420261282797).

### Search strategy

A systematic electronic search was performed in the PubMed, Scopus, Nature, MedRxiv, bioRxiv, databases to identify studies on the use of AI algorithms for predicting 5-year overall survival (5-OS) in patients who underwent surgical treatment for gastric cancer, based on clinical data from January 2015 to July 2025. The search in MedRxiv and bioRxiv did not identify any articles for inclusion in the analysis. The search strategy is detailed in (Supplementary Table S1).

### Eligibility criteria

Study eligibility criteria for this systematic review were based on the population, intervention, comparisons, outcomes, settings, and study designs of interest (PICOS) framework. The population of interest was adults over 18 with histologically confirmed gastric cancer who underwent surgery with radical intent. We have included studies using AI models investigating 5-year OS based on data features extracted from EHRs. Details on the PICOS selection criteria are summarized in (Table 1).

**Table 1:** PICOS criteria and exclusion criteria.

Inclusion criteria (PICOS)	Exclusion criteria
Population: Age ≥18 years histologically confirmed gastric cancer radical surgery	Reporting model accuracy for 5-OS only within stratified subgroups (clustering) rather than for the overall cohort.
Interventions: An AI model built and trained to predict 5-OS based on patient clinical data.	Absence of AI application for direct 5-OS prediction, including constructing 5-OS nomograms from data derived from AI models predicting recurrence-free survival.
Comparators: Machine learning models versus conventional statistical methods for 5-OS prediction, and performance comparison among different machine learning algorithms	Use of parameters in AI model training that requires specialized approaches (histoscan analysis, radiomics, non-routine blood metabolite assays, and genomics).
Outcomes: Model performance assessment by mean AUC value.	Absence of results presented as AUC.
Study design: Retrospective single-center and multi-center studies.	Absence of specific 5-OS results despite having generalized OS prediction results (without a specific time interval).

Table 1 outlines the predefined PICOS framework used to guide study eligibility, detailing the inclusion and exclusion criteria that structured the selection of publications for this systematic review.

### Data extraction

Two reviewers (MM and ZS) performed an independent review of publications. Duplicates were removed, and article titles were screened. Full-text articles with relevant titles and abstracts were shortlisted. Each shortlisted article was then assessed for eligibility based on inclusion and exclusion criteria for direct inclusion or exclusion from the systematic review. The decision to include an article was made by two reviewers; in case of disagreement, a third reviewer (GS) made the final decision. Reasons for exclusion were documented and are presented in the “Results” section in the flow diagram. The following data were extracted from the included studies: first author and year of publication, country of origin, study design (single-center or multicenter), validation methodology (internal, external, or cross-validation), patient eligibility criteria, demographic characteristics (sex, age), disease stage (TNM classification), machine learning algorithms employed, conventional statistical comparator models (when applicable), number of features selected for model development, and feature selection methodology.

### Quality assessment

Two independent authors performed the quality assessment (AR and BI). Any differences in assessment were discussed with the senior author (GS) until a mutual agreement was reached. To assess the quality of included studies, the risk of bias was assessed using the PROBAST-AI (4).

### Statistical analysis

The evaluated outcome was the mean AUC with 95% confidence interval (CI) for machine learning-based models compared with conventional statistical approaches. Pooled measures with 95% CIs were calculated for individual groups. A result was considered statistically significant at a *p*-value < 0.05. When heterogeneity between studies was high, a random-effects model was used to plot the graphs. When heterogeneity was below 25%, a fixed-effects model was used.

Heterogeneity was assessed using I², where values of 0%–25% indicated low heterogeneity, 25%–75% moderate, and above 75% high heterogeneity. Analysis was performed using Review Manager Version 5.4.1. The model for constructing forest plots was selected as “Mean,” allowing comparison of mean values of different metrics. When studies lacked data on the confidence interval for the AUC result, it was calculated using the formula from Hanley & McNeil (5). Subgroup analyses were performed given a sufficient number of comparable metrics.

## Results

### Search and study characteristics

**Figure 1:**
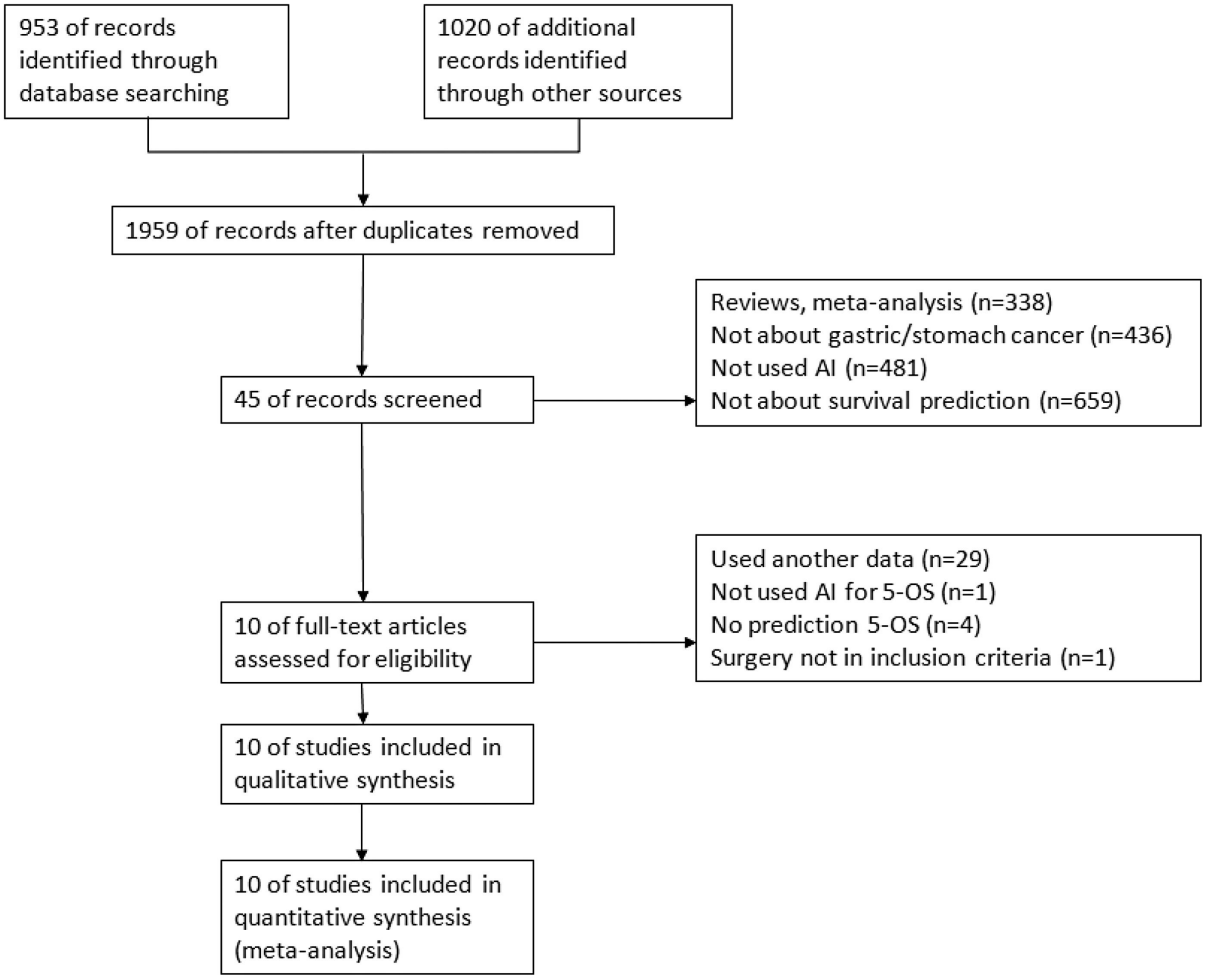
Flow diagram.

The literature search using the above query yielded 1,959 articles. Full texts of 45 articles were reviewed, and 10 articles were included in the final analysis (Figure 1). All included articles pertained to retrospective studies, of which 6 (60%) were multi-center and 4 (40%) were single-center. Six (60%) studies had external validation, while 4 (40%) had internal or cross-validation. All studies were conducted in Asian countries: 8 (80%) in China, 1 (10%) in Japan, and 1 (10%) in Korea (Table 2 summarizes the studies included in the meta-analysis).

**Table 2:** Summary of included studies.

Study	Year	Study design/centers	Sample size	Data features	Models compared	Performance (AUC, 95% CI)	Feature selection/notes
Li et al. (6)	2022	Multicenter	543 external; 43 internal	9	SVM vs. TNM	External SVM: 0.826 (0.779–0.873); Internal TNM: 0.617 (0.472–0.762); Internal SVM: 0.706 (0.547–0.865)	Prognostic factors selected from comprehensive clinical information
Kuwayama et al. (7)	2023	Single-center	1,687	10	LR, RF, GBM, DNN	LR: 0.702 (0.670–0.734); RF: 0.721 (0.695–0.747); GBM: 0.730 (0.704–0.756); DNN: 0.682 (0.650–0.714)	K-medoids method
Chung et al. (8)	2023	Multicenter	4,025 training cohort	28	XGBoost, RF, GBM, AdaBoost, LightGBM, CatBoost	XGBoost: 0.8118 (0.7971–0.8265); RF: 0.7741 (0.7393–0.8089); GBM: 0.7846 (0.7491–0.8201); AdaBoost: 0.7844 (0.7409–0.8279); LightGBM: 0.7846 (0.7404–0.8288); CatBoost: 0.7932 (0.7556–0.8308)	K-fold cross-validation
Wu et al. (9)	2024	Multicenter	2,846 external validation cohort	11	DL, Multitask LR, TNM, RF	DL: 0.750 (0.732–0.768); Multitask LR: 0.740 (0.722–0.758); TNM: 0.670 (0.651–0.689); RF: 0.700 (0.681–0.719)	Univariate overall survival analysis; Kaplan–Meier survival analysis
Li et al. (10)	2020	Multicenter	1,432 external validation cohort	11	ANN vs. TNM	ANN: 0.850 (0.826–0.874); TNM: 0.821 (0.795–0.847)	LASSO Cox regression model
Zhan et al. (11)	2024	Multicenter	228	19	ANN, SVM, RF, LR, GBM, CatBoost, KNN, Decision Tree	ANN: 0.782 (0.757–0.807); SVM: 0.786 (0.749–0.823); RF: 0.728 (0.675–0.781); LR: 0.793 (0.762–0.824); GBM: 0.715 (0.652–0.778); CatBoost: 0.757 (0.697–0.817); KNN: 0.745 (0.705–0.785); Decision Tree: 0.631 (0.545–0.717)	Recursive feature elimination (RFE)
Zhang et al. (12)	2025	Multicenter	492 external validation cohort	6	RF, SVM, DT	RF: 0.796 (0.755–0.837); SVM: 0.789 (0.748–0.830); DT: 0.741 (0.699–0.783)	X-tile; Kaplan–Meier survival analyses
Ji et al. (13)	2024	Single-center	431 intestinal type; 217 diffuse type	5 intestinal; 8 diffuse	RF, GBDT, DT, KNN, XGBoost, LR, SVM	Intestinal type — RF: 0.815 (0.775–0.855); GBDT: 0.822 (0.783–0.861); DT: 0.790 (0.748–0.832); KNN: 0.705 (0.656–0.754); XGBoost: 0.810 (0.769–0.851); LR: 0.740 (0.694–0.786); SVM: 0.690 (0.641–0.739). Diffuse type — RF: 0.840 (0.783–0.897); GBDT: 0.878 (0.828–0.928); DT: 0.860 (0.806–0.914); KNN: 0.710 (0.639–0.781); XGBoost: 0.870 (0.818–0.922); LR: 0.670 (0.596–0.744); SVM: 0.810 (0.749–0.871)	RFE via 10-fold cross-validation and joint LASSO screening
Liu et al. (14)	2022	Single-center	955	4	Machine learning vs. LASSO	Machine learning: 0.800 (0.772–0.828); LASSO: 0.800 (0.772–0.828)	Univariate Cox regression model; candidate algorithms included Boruta, Elastic Net, SVM, and Random Forest
Zeng et al. (15)	2024	Multicenter	3,287	16	DeepSurv, Cox model, RF	DeepSurv: 0.868 (0.854–0.882); Cox model: 0.796 (0.779–0.813); RF: 0.793 (0.776–0.810)	Univariate and multivariate Cox regression analysis; K-fold validation

Table 2 presents the main characteristics of the studies included in the meta-analysis, summarizing key methodological features such as sample size, type of validation, AI architectures employed, and performance metrics used by the respective authors.

Most studies reported discrimination metrics and used validation methods such as cross-validation or train-test splitting, but only two reported calibration plots and few presented decision curve analysis. Feature selection was common, using methods from clinical judgment to LASSO and recursive feature elimination. However, model explainability was rarely addressed: only recent studies used SHAP analysis, while others provided limited or no interpretability assessment (Supplementary Table S2.3).

### Quality assessment of included studies

**Figure 2:**
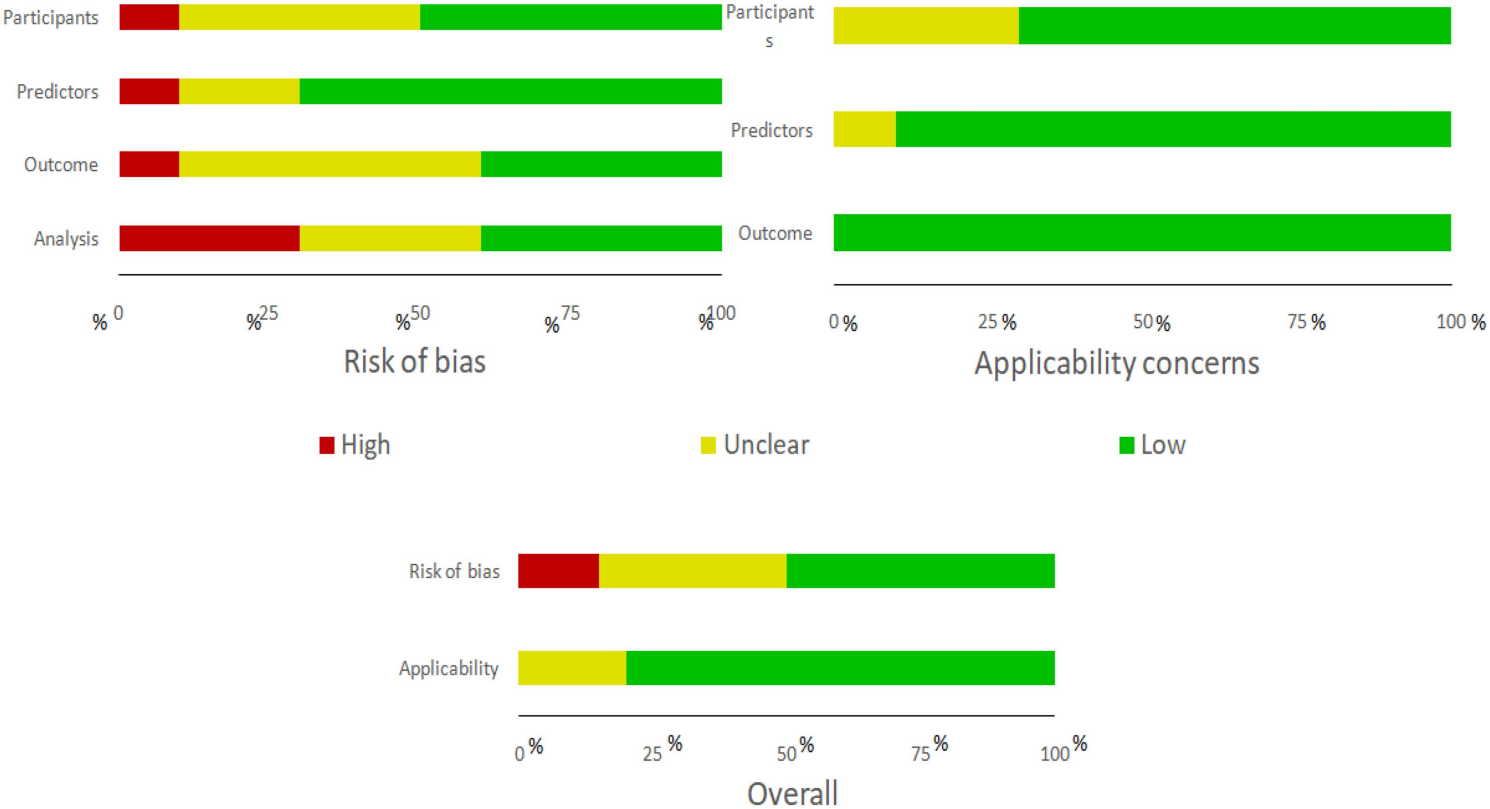
Quality assessment by PROBAST-AI.

In the domain of analysis, three out of ten studies (30%) were rated as having a high risk of bias, three (30%) as having an unclear risk, and four (40%) as having a low risk. In the other three domains, only one study was rated as high risk in each, resulting in a proportion not exceeding 10% per domain. In the outcome domain, five studies (50%) were judged to have an unclear risk of bias. Each domain contained at least four studies (40%) with a low risk of bias. The domain of predictors had the lowest risk, with seven studies (70%) rated as low risk. The assessment of applicability concerns did not identify any studies at high risk in any domain. All studies were deemed to have low applicability concerns for the outcome domain. Concerns were raised in two domains: one study (10%) was rated as unclear in the predictors domain, and three studies (30%) were rated as unclear in the participants domain. Despite the identified information gaps, it was decided not to exclude any study from the subsequent meta-analysis, as the noted presentation and methodological nuances likely do not critically impact the meta-analysis results, and the overall bias for each criterion did not exceed 50%.The summarized data on the results of the study quality assessment are presented in Figure 2.

Full details of the methodological heterogeneity across studies are presented in Supplementary Table S2.1 in the Supplement.

### Patient characteristics

A total of 15,643 patients from cohorts whose data were considered in this meta-analysis (including results for cohorts with a comparison group for the set task or external cohorts if a comparison group was present for all) were included. Of these, 10,311 (65.9%) were male and 5,104 (32.6%) were female. The study by Zhan Z was not included in the sex distribution analysis due to presentation of aggregate data for both training and test sets without comparison. The mean patient age was 61.7 years. Analysis by stage showed: 5,852 (37.4%) patients had initial stage I disease, 8,452 (54%) had stage II-III, and 1,054 (6.7%) had stage IV. The Zhan Z study was not included in stage distribution analysis for the reason above, and in the Li Z study, 84 (5.7%) patients lacked staging data. Full details of the clinical heterogeneity across studies are presented in Supplementary Table S2.2 in the Appendix.

### Characteristics of applied AI algorithms

The most frequently used AI algorithm was RF (Random Forest), employed in 7 (70%) of the studies. Various boosting algorithms, such as GBM, AdaBoost, LightGBM, CatBoost, and XGBoost, were applied in 4 (40%) of the studies. Algorithms based on artificial neural networks (ANNs) were used in 5 (50%) of the studies, with deep learning applied in 4 (40%) and machine learning in 1 (10%). Logistic Regression (LR) was utilized in 4 (40%) of the studies. The Support Vector Machine (SVM) algorithm was used in 4 (40%) of the studies. The Decision Tree algorithm was used in 3 (30%) of the studies, and K-Nearest Neighbors (KNN) in 2 (20%). More than one AI algorithm was used for 5-OS prediction in 7 (70%) of the studies. **Funnel plots** presented in Supplementary Figures S3.1-S7.1 in the Supplement.

### Machine learning models versus traditional statistical methods

**Figure 3:**
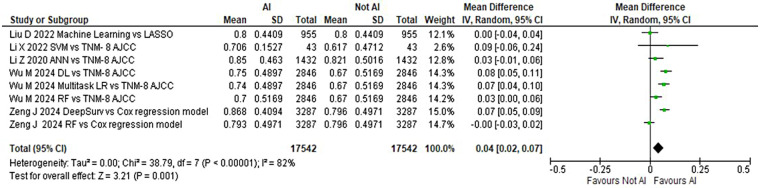
Forest plot: ML models vs. conventional statistical models.

Among the included studies, 5 compared the effectiveness of AI-based models with other predictive methods such as Cox regression, LASSO regression, and TNM staging. Our meta-analysis included 5 studies and 8 metrics because two studies compared multiple AI models against a standard predictive method. The primary reference method was TNM staging (3 studies), Cox model (1 study), and LASSO regression (1 study). High heterogeneity was observed (I²=82%). Nevertheless, machine learning-based models demonstrated a modest but statistically significant improvement in predictive performance compared with conventional statistical approaches, with a pooled mean AUC increase of 0.04 (95% CI 0.02–0.07; *p* = 0.001) (Figure 3).

Given the high heterogeneity, future validation on a larger number of publications is required.

### Results of comparison: “bagging” vs. “boosting” AI algorithms

**Figure 4:**
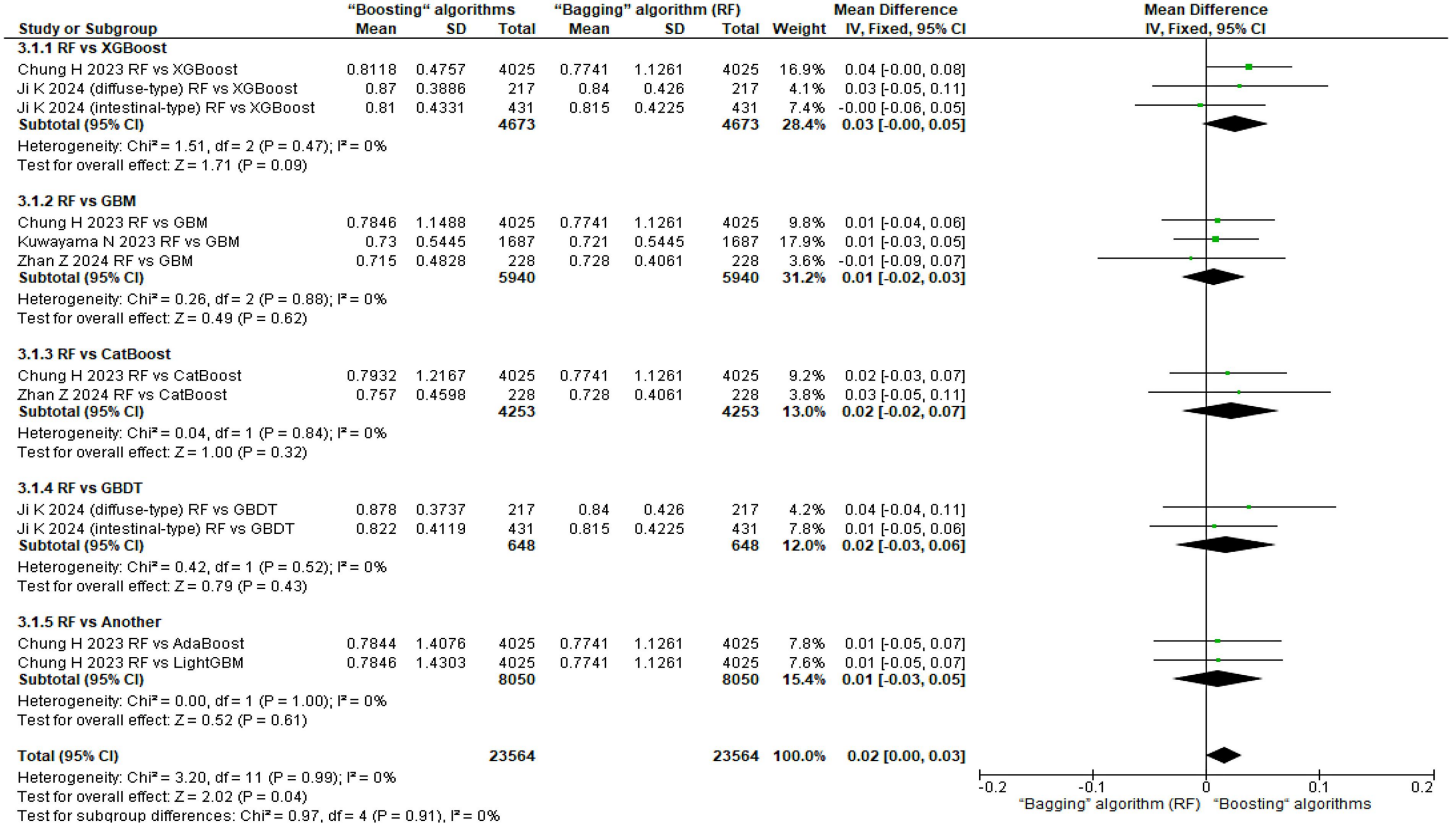
Forest plot «bagging» vs. «boosting» algorithms.

As noted, RF was the most used algorithm. Boosting algorithms were used in 40% of studies, and in all 4 studies using boosting algorithms, RF was also used. We performed a meta-analysis comparing the effectiveness of RF (conventionally a “bagging” algorithm) and various boosting algorithms. Heterogeneity was low (I² = 0%). Subgroup analyses comparing RF with specific boosting types showed no statistically significant difference. However, the overall meta-analysis result showed a statistically significant (*p* = 0.04) improvement in AUC with the use of boosting algorithms (Figure 4). Therefore, using both types of ensemble algorithms in a single study of similar design is likely unnecessary due to highly comparable results. If choosing one, preference might be given to boosting algorithms given the statistically significant, albeit slight, improvement.

### Results of comparison: RF vs. SVM algorithms

**Figure 5:**
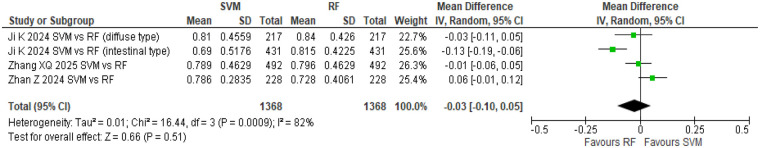
Forest plot SVM vs. RF.

A meta-analysis of AUC results for RF and SVM (another frequently used algorithm) was performed. Heterogeneity was high (I² = 82%). No statistically significant difference was found (*p* = 0.51) (Figure 5). More publications and deeper analysis of criteria for choosing between RF and SVM are likely needed.

### Results of comparison: RF vs. neural network algorithms

**Figure 6:**
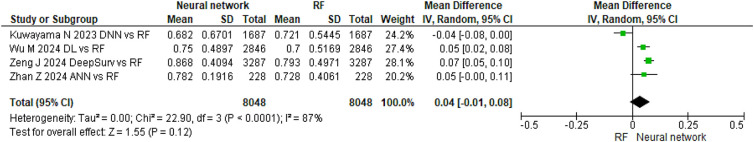
Forest plot RF vs. Neural network algorithms.

A meta-analysis comparing RF and neural network algorithms was conducted. High heterogeneity was found (I² = 87%), and no statistically significant improvement in mean AUC [0.04 (95% CI −0.01, 0.08)] was detected (*p* = 0.12) (Figure 6).

**Figure 7:**
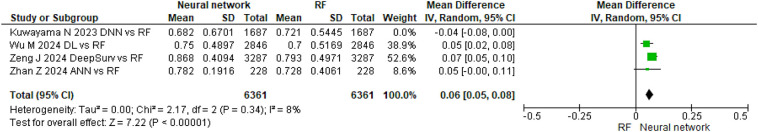
Forest plot RF vs. Neural network algorithms (without Kuwayama N study).

However, upon excluding the Kuwayama N study, heterogeneity dropped to I² = 8%, and the meta-analysis showed a statistically significant (*p* < 0.00001) and most pronounced increase in mean AUC [by 0.06 (95% CI 0.05, 0.08)] for neural networks compared to RF (Figure 7).

### Results of comparison: RF vs. LR algorithms

**Figure 8:**
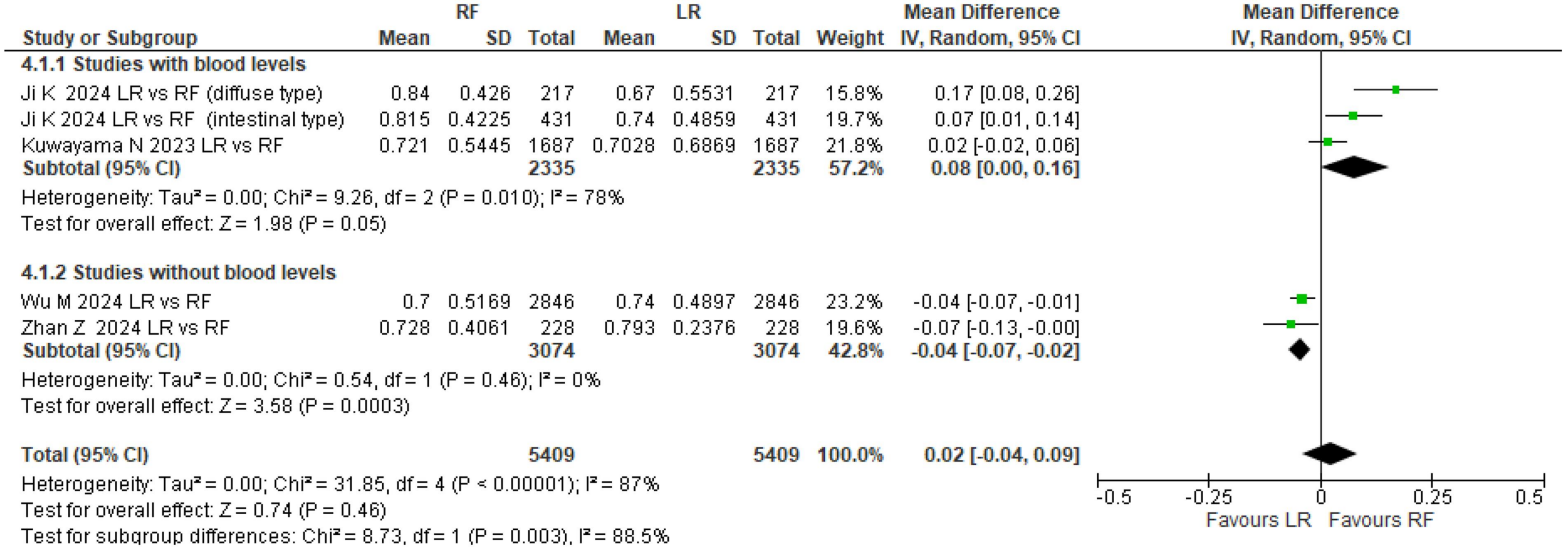
Forest plot RF vs. LR.

Another common algorithm was LR. Analysis revealed significant variation in AUC differences when comparing RF and LR. We separately evaluated studies using both algorithms and, considering the hypothesis that inclusion of blood parameters might affect results, divided the 4 studies into two subgroups: studies without blood parameters and studies with blood parameters. The subgroup analysis proved accurate, yielding opposite results. In the subgroup using blood parameters, high heterogeneity (I² = 78%) and increase in mean AUC for RF over LR [0.08 (95% CI 0.00, 0.16)] was observed. In the subgroup without blood parameters, low heterogeneity (I² = 0%) and a statistically significant (*p* = 0.0003) increase in mean AUC for LR over RF [0.04 (95% CI −0.07, −0.02)] was found (Figure 8). Thus, the nature of selected clinical data may influence the choice of AI algorithm.

### Clinical variables used in the studies

Clinical features used for AI training were analyzed. A list of the most important features according to the study authors (where available) is presented in Table 2. In 50% of the studies, important features were age, T-stage, and tumor size. In 40% of studies, prognostic value was also found for: serum albumin level (or prealbumin) and the ratio of metastatic to examined lymph nodes (mLN ratio). Features such as tumor markers and tumor grade (G) were used more limitedly (significant in 20% and 10% of studies, respectively).

Table 3 synthesizes the clinical input variables utilized for AI model development in the included studies, emphasizing the frequency and prognostic relevance of specific features such as age, tumor stage, tumor size, and serum biomarkers.

**Table 3:** Clinical variables used in AI models.

Kuwayama et al. (2023) (7)	Chung et al. (2023) (8)	Li et al. (2020) (10)	Zhan et al. (2024) (11)	Zhang et al. (2025) (12)	Ji et al. ( 2024) (13) (intestinal type)	Ji et al. (2024) (13) (diffuse type)	Liu et al. (2022) (14)	Zeng et al. (2024) (15)
Age	Age	Depth of invasion	mLN ratio	ALB	Stage	Stage	mLN ratio	mLN ratio
ALB	Preoperative albumin	number of mLN	T stage	TBIL	CA-125	Borrmann type IV	Age	Age
CEA	Preoperative NRI	Age	Tumor size	Blood ureanitrogen (BUN)	Tumor size	Lower LYM	Regional nodes examined (RNE)	T stage
CA 19-9	T stage	mLN ratio	Resection margins		CA 19-9	Higher LDH	T stage	Chemotherapy
Hematocrit (Hct)	Difference in the percentage of hemoglobin	Tumor size	PNI		Lower serum prealbumin (PALB)	Lower K	Grade	
Hemoglobinlevel (Hb)	Stage					Positive PNI		
Prothrombintime (PT)	1 year postoperative NRI					Tumor size		
Platelet (PLT)	Tumour size							
	Number of metastatic lymph nodes							

## Discussion

This study analyzed 10 research articles investigating the role of artificial intelligence (AI) in predicting 5-year overall survival (5-OS) based on clinical data for patients who underwent surgical treatment for gastric cancer. An assessment of clinical and methodological heterogeneity revealed no critical differences between the studies, and therefore, all were included in the subsequent meta-analysis.

The initial analytical phase compared the Area Under the Curve (AUC) values of prognostic models utilizing AI algorithms against those employing no AI methodology. For this comparison, data from 5 out of the 10 selected studies were included.

Substantial heterogeneity was observed among studies (I² = 82%). Nevertheless, machine learning-based models demonstrated a modest but statistically significant improvement in predictive performance compared with conventional statistical approaches, with a pooled mean AUC increase of 0.04 (95% CI 0.02–0.07; *p* = 0.001) for 5-OS prediction. The most favorable result was reported in the study by Wu M., which compared deep learning algorithms against TNM staging, whereas the least favorable outcome was observed for the Random Forest (RF) algorithm compared to Cox regression.

Given the limited number of studies and their substantial heterogeneity, future validation of the observed improvement in mean AUC with AI application is warranted. These findings indicate that machine learning models generally outperform conventional statistical approaches for 5-OS prediction. However, algorithm selection should be guided by the specific clinical data structure, the comparator method, and the machine learning architecture employed. For instance, while TNM staging evidently possesses the lowest predictive value, employing Cox regression or logistic regression on categorical data may, in theory, yield results comparable to or, in some cases, even surpass those of simple AI algorithms.

In the second phase of our work, we performed a comparative analysis of various artificial intelligence (AI) algorithms. The most frequently employed AI algorithm in studies focused on predicting 5-year overall survival (5-OS) in patients undergoing surgical treatment for gastric cancer is Random Forest (RF). Furthermore, “boosting” algorithms were utilized in nearly half of the cases (40%), with all four studies that implemented “boosting” algorithms also employing RF. Both RF and “boosting” algorithms belong to the class of ensemble algorithms.

Ensemble algorithms constitute a machine learning methodology that combines multiple base models to create a single, more predictive model. In “bagging” algorithms, which conventionally include RF, models are trained independently of one another, typically in parallel on different subsets of data. Conversely, in “boosting” algorithms, models are trained sequentially.

We conducted a meta-analysis to compare the predictive efficacy for 5-OS between RF (conventionally designated as a “bagging” algorithm) and various “boosting” algorithms. This comparison aimed to assess the rationale for their combined application within a single study when developing AI models based on clinical data for 5-OS prediction. Heterogeneity among the included studies was determined to be low (I² = 0%). Subgroup analyses comparing RF with specific types of “boosting” algorithms did not reveal any statistically significant differences in predictive performance as measured by the Area Under the Curve (AUC). However, the overall meta-analysis result demonstrated a statistically significant (*p* = 0.04) improvement in the AUC metric by 0.02 favoring “boosting” algorithms.

Consequently, the simultaneous application of both distinct types of ensemble algorithms in the planning of studies with a similar design appears to be weakly justified from both economic and practical standpoints, given the anticipated high degree of comparability in outcomes. Should a single algorithm be selected, preference should be accorded to “boosting” algorithms, as the observed improvement in AUC, albeit marginal, attained statistical significance.

Furthermore, the Support Vector Machine (SVM) algorithm was utilized in 40% of the analyzed studies. SVM is a supervised machine learning algorithm designed for binary classification. Its objective is to identify an optimal hyperplane that maximizes the margin—the distance—between the nearest data points of opposing classes (support vectors). The dimensionality of this hyperplane is contingent upon the number of features in the input data, manifesting as a line in two-dimensional space or a plane in n-dimensional space. While multiple separating hyperplanes may exist, the algorithm's optimization criterion specifically selects the one that provides the maximal margin, thereby determining the optimal decision boundary.

The analysis indicated a non-significant trend favoring Random Forest (RF), with a mean increase in the Area Under the Curve (AUC) of 0.03 (95% CI: −0.10, 0.05). However, the overall mean difference in AUC for 5-year overall survival (5-OS) prediction between RF and SVM models was not statistically significant (*p* = 0.51).

Within this systematic review, a meta-analysis was also conducted to compare the predictive efficacy, measured by AUC, of models based on RF and Neural Networks (NN), the latter representing a highly promising direction in AI. A high degree of heterogeneity was observed among the included publications (I² = 87%), with no statistically significant improvement in the mean AUC for NN over RF (mean difference: 0.04, 95% CI: −0.01, 0.08; *p* = 0.12). Notably, upon exclusion of the study by Kuwayama N. et al., heterogeneity markedly decreased (I² = 8%), and the meta-analysis yielded a statistically significant (*p* < 0.00001) and pronounced increase in the mean AUC of 0.06 (95% CI: 0.05, 0.08) in favor of NN models. The most plausible explanation for this discrepancy is that the study by Kuwayama N. et al. was the only one among the four included that incorporated blood-based biomarkers and was also the earliest publication (2023). Nevertheless, this hypothesis cannot be definitively confirmed or refuted within the scope of the present investigation.

Logistic Regression (LR) was another widely employed algorithm, featured in 40% of the studies included in this systematic review. LR is a supervised learning algorithm that models the relationship between a categorical dependent variable and one or more independent variables, proving particularly useful for binary outcomes. Initial data analysis revealed considerable variability in the AUC difference when comparing RF and LR. Given the intrinsic characteristics of LR and our hypothesis that the nature of the clinical data directly influences algorithmic performance, studies utilizing both RF and LR were stratified based on the inclusion or exclusion of blood-based parameters. In studies incorporating blood biomarkers, RF demonstrated a mean AUC increase of 0.08 compared to LR (95% CI: 0.00, 0.16; *p* = 0.05). Conversely, in studies excluding blood parameters, LR exhibited a modest but statistically significant mean AUC advantage of 0.04 (95% CI: −0.07, −0.02; *p* = 0.0003). This divergent performance is likely attributable to the data characteristics.

LR typically handles categorical data effectively but may underperform with high-dimensional or complex numerical data. In contrast, ensemble algorithms like RF are generally robust in processing quantitative data but are susceptible to overfitting when dealing with sparse categorical features. Consequently, the performance (AUC) of a predictive model depends not only on the implementation of an AI-based method but also critically on the nature of the input data and the specific AI algorithm selected to match that data structure. We posit that this is one of the most significant findings of this work, as a data-type-driven approach to AI algorithm selection in the planning phase of prognostic model development can optimize resource allocation while maximizing predictive accuracy.

In studies providing accessible information on feature importance or a ranked list of included clinical indicators, the frequency of their occurrence across each research article was analyzed. The most consistently significant prognostic indicators identified were: patient age, T stage, tumor size, serum albumin (or prealbumin) level, and the metastatic-to-examined lymph node ratio (mLNR). These variables may warrant more focused attention in future research for the development and training of models that explicitly incorporate each of them.

Furthermore, the findings from this work suggest that the T category of the TNM staging system possesses substantial prognostic power for 5-year overall survival (5-OS). In contrast, the assessment of the N category, which is based solely on the number of metastatic lymph nodes, appears to have a lower prognostic utility. This implies that the mLNR might serve as a more effective predictor and could potentially be considered as a superior alternative in future prognostic modeling compared to the conventional N category.

Investigating the role of artificial intelligence (AI) in predicting gastric cancer survival remains a highly relevant research focus. Notably, in 2025, Wang H.N. et al. published a systematic review analyzing the potential of machine learning for survival prediction in gastric cancer. Their literature search yielded 16 studies for inclusion. The most frequently employed algorithms identified in that review were deep learning (37.5%), Random Forest (RF) (37.5%), Support Vector Machine (SVM) (31.25%), and ensemble methods (18.75%). The reported Area Under the Curve (AUC) values ranged from 0.669 to 0.980 for overall survival, 0.920–0.960 for cancer-specific survival, and 0.710–0.856 for relapse-free survival (2). Thus, that study also underscores the growing role of AI in predicting gastric cancer outcomes. The principal distinctions of our work from the aforementioned publication are: exclusive focus on 5-OS, inclusion of studies only with available AUC metrics, analysis restricted to models using readily accessible clinical data, and the execution of head-to-head meta-analyses. Nevertheless, the synthesized conclusion is congruent—the application of AI enhances survival prediction capabilities.

Our study has several limitations. Firstly, it was conducted on a limited number of publications due to the specificity of the research question. Secondly, high heterogeneity was observed in several of the analyses performed. The high heterogeneity of AI-based prognostic studies is a major issue, limiting the interpretability of other meta-analyses (16). Even though we've focused our research focus on EHR-based prognostic models, we still observed a high heterogeneity across included studies. The “black-box” nature of machine learning and deep learning algorithms and the complexity of prognostic models development may further limit the reproducibility of these results.

Thirdly, all included studies were conducted in Asian countries; therefore, the generalizability of the findings to European patient cohorts requires careful consideration and further validation. Additionally, it should be noted that 30% of the studies lacked external validation. Furthermore, 40% of the studies utilized data from publicly available databases (one study used a TCGA cohort for external validation, and three studies employed SEER data), which may introduce bias, potentially leading to an artificial inflation or deflation of the achievable AUC. Consequently, confirmatory studies are necessary to substantiate the results presented here.

An additional important finding of this review is the methodological imbalance across the included machine learning studies. While discrimination was almost universally reported, calibration, explainability, and broader clinical utility assessment were much less consistently addressed. This is important because a model with a favorable AUC may still be poorly calibrated or difficult to interpret in routine clinical use. In the present evidence base, feature selection and internal validation were relatively common, but formal calibration reporting was limited to a small subset of studies, and advanced explainability methods such as SHAP were used only in more publications that are recent. These observations suggest that the current literature may overemphasize discrimination performance while underreporting the properties that are most relevant for implementation, reproducibility, and clinician trust.

## Conclusion

AI-based prognostic models utilizing routinely available clinical data provide clinically meaningful improvements in 5-year overall survival prediction after gastric cancer surgery. This systematic review and meta-analysis demonstrates that the performance of AI algorithms depends on the structure and type of input data, particularly the inclusion of blood-based biomarkers. Selection of AI models guided by data characteristics may improve both predictive accuracy and practical applicability in clinical decision support systems.

## Data Availability

The original contributions presented in the study are included in the article/Supplementary Material, further inquiries can be directed to the corresponding author.
