# Correction: Gastric cancer survival prediction using artificial intelligence models based on electronic health records: a systematic review and meta-analysis

**DOI:** 10.3389/fdgth.2026.1920862

**Published:** 2026-07-09

**Authors:** Maryana Mandrina, Tigran Gevorkyan, Sergey Zvezda, Valeria Pavlova, Rukiyat Abdulaeva, Mariam Manukyan, Yana Belenkaya, Sergey Gordeyev, Ivan Stilidi

**Affiliations:** 1 N.N. Blokhin National Medical Research Center of Oncology, Moscow, Russia; 2 Tyumen State Medical University, Tyumen, Russia; 3 Sechenov University, Moscow, Russia

**Keywords:** artificial intelligence, electronic health records, gastric cancer, meta-analysis, survival prediction, systematic review

An incorrect Funding statement was provided. The correct funding statement is “The author(s) declared that financial support was received for this work and/or its publication. This work was funded by The Ministry of Economic Development of the Russian Federation (Grant No. 139-15-2025-008) in the form of a subsidy from the Federal Budget to the Federal State Budgetary Institution ‘N.N. Blokhin National Medical Research Center of Oncology’ of the Ministry of Health of the Russian Federation, State Contract ID 000000С313925P3R0002 dated 16 April 2025.”

The original version of this article has been updated.

