# Supplementary material for: Gastric cancer survival prediction using artificial intelligence models based on electronic health records: a systematic review and meta-analysis
Source: Front Digit Health. 2026 Jun 23;8:1845932. doi: 10.3389/fdgth.2026.1845932 (PMC13337829; doi:10.3389/fdgth.2026.1845932)
Supplement: Supplementary file 1 [file Datasheet1.docx]

**Supplementary Table S1. Search strategy**

| Pubmed | ("Artificial Intelligence"[MeSH Terms] OR "Machine Learning" OR "Deep Learning" OR "Artificial Intelligence"[Title/Abstract] OR "Machine Learning"[Title/Abstract] OR "Deep Learning"[Title/Abstract]) AND ("Stomach Neoplasms"[MeSH Terms] OR "Gastric Cancer"[Title/Abstract] OR "Stomach Cancer"[Title/Abstract] OR "Gastric Carcinoma"[Title/Abstract]) AND ("Prognos*" OR "Predict*" OR "Survival"[Title/Abstract] OR "Overall Survival" OR "Outcome*" OR "Risk Prediction" OR "Risk Stratification") |
| --- | --- |
| Scopus | TITLE-ABS-KEY ( ( "Artificial Intelligence" OR "Machine Learning" OR "Deep Learning" OR "Neural Network*" OR "Supervised Learning" OR "Unsupervised Learning" )  AND  ( "Gastric Cancer" OR "Stomach Cancer" OR "Gastric Carcinoma" OR "Stomach Neoplasm*" )  AND  ( prognos* OR predict* OR "survival" OR "overall survival" OR "disease-free survival" OR "recurrence" OR "outcome*" OR "risk prediction" OR "risk stratification" OR "survival prediction" ) ). |
| MedRxiv, bioRxiv | ("Artificial Intelligence" OR "Machine Learning" OR "Deep Learning") AND ("Gastric Cancer" OR "Stomach Cancer") AND (prognos* OR predict* OR survival OR "overall survival") |

Supplementary Table S1 describes the exact query strings applied across multiple databases, enabling replication of the systematic search procedure.

**Supplementary Table S2.1 - Methodological heterogeneity of the included studies**

| **Author** | **Year** | **Type of study** | **AI algorithm** | **Number of patients** | **Inclusion criteria** | **Exclusion criteria** | **Validation** |
| --- | --- | --- | --- | --- | --- | --- | --- |
| 1. Zeng J | 2024 | Retrospective single-center | DeepSurv  AUC  0.868 (95% CI 0.854, 0,882)  RF  AUC  0.793 (95% CI 0.776, 0,81 | 3287 | 1. Localization and morphological code "stomach";  2. Histological codes, including 8140/3, 8141/3, 8142/3, 8143/3, 8144/3, 8262/3, and 8323/3 [International Classification of Diseases for Oncology, Third Revision (ICD-O-3)]  3. Pathologically confirmed malignant tumor  4. Age over 20 years.  5. History of surgical treatment | 1. Lack of demographic information such as gender, marital status, and race;  2. Information on cause of death or subsequent survival was unavailable;  3. Patients who lack clinical knowledge such as histopathological information, surgical information, primary tumor site code, T stage (AJCC stage 7), N stage (AJCC stage 7), or clinical grade. | External |
| 2. Liu D | 2022 | Retrospective single-center | Machine Learning  AUC  0.800 (95% CI 0.772, 0,828) | 955 | 1. Surgical intervention  2. Gastric adenocarcinoma | 1. Lack of complete data | External |
| 3. Li X | 2022 | Retrospective multicenter | SVM  AUC 0.706 (95% CICI 0,547–0,865) | 43 | Surgical resection R0 | 1. Preoperative chemotherapy  2. R1/R2 resection  3. Serious surgical complications  4. Lack of complete follow-up data for 3 years  5. Presence of distant metastases  6. MPMN | External |
| 4. Wu M | 2024 | Retrospective multicenter | DL  AUC  0.750 (95% CI 0.732–0,768)  Multitask LR  AUC 0.740  (95% CI 0.722, 0,758)  RF  AUC 0.700  (95% CI0.681, 0,719) | 2846 | 1. Age over 18  2. Surgical intervention | 1. Individuals with unknown age 2. Unknown survival time | External |
| 5. Li Z | 2020 | Retrospective multicenter | ANN  AUC  0.850 (95% CI, 0.826‐0.874) | 1432 | 1. Primary gastric cancer with R0 resection  2. Adenocarcinoma (papillary, tubular, poorly differentiated, mucinous, and signet-ring cell carcinoma)  3. No distant metastases | Lack of complete data | External |
| 6. Zhan Z | 2024 | Retrospective multicenter | ANN  SVM  RF  LR  GBM  CatBoost  GNB  KNN  DecisionTree | 228 | 1. Surgical treatment, including radical and palliative surgery  2. Follow-up duration > 5 years or death | 1. Neoadjuvant therapy  2. R1/R2 resection after previous gastrectomy, 3. MPMN within the last 5 years  4. Death within 3 months after surgery,  5. Not adenocarcinomas  6. Incomplete clinical and pathological data. | Internal |
| 7. Ji K | 2024 | Retrospective single-center | LR  SVM  RF  GBDT  DT  KNN  XGBoost | 648 | 1. Confirmation of gastric cancer by tissue biopsy and postoperative pathological examination;  2. Pathological confirmation of intestinal or diffuse gastric cancer; 3. Surgical treatment for gastric cancer;  4. Availability of complete clinical and pathological data or absence of less than 30% of clinical and pathological data. | 1. Previous treatment with neoadjuvant radiotherapy and/or chemotherapy  2. History of gastric cancer recurrence. | Internal |
| 8. Zhang XQ | 2025 | Retrospective multicenter | SVM  RF  DT | 492 | 1. Biopsy-confirmed gastric adenocarcinoma  2. Age at diagnosis ≥ 65 years  3. Radical surgical resection  4. Availability of complete clinical and pathological data. | . 1. Postoperative pathology confirming non-gastric primary tumors  2. Distant metastases  3. Incomplete clinical data  4. Detection of other concomitant malignancies within five years. | External |
| 9. Kuwayama N | 2023 | Retrospective single-center | LR  RF  GBM  DNN | 1687 | Surgicalintervention | Notspecified | Not specified |
| 10. Chung H | 2023 | Retrospective multicenter | XGBoost  GBM  AdaBoost  LightGBM  CatBoost  RF | 4025 | Surgicalintervention | Death within 1 year after surgery | External |

Supplementary Table S2.1 outlines sources of methodological heterogeneity among the included studies, providing details on cohort design, validation frameworks, and criteria that may influence model comparability.

**Supplementary Table S2.2 -** Clinical heterogeneity of included studies (Appendix)

| **Study** | **Middle age** | **Men** | **Women** | **Istage** | **II-III**  **stages** | **IV**  **stage** |
| --- | --- | --- | --- | --- | --- | --- |
| 1. Zeng J | 20-69  1727(52.54%)  70+  1560(47.46%) | 2276(69.24) | 1011(30,76) | 1002(30.48) | 1873 (56.98) | 412(12.53) |
| 2. Liu D | <70 149(15. 6)  ≥70 806 (84,3) | 263 (27. 6) | 692 (72,4) | 219 (22.9) | 623  (65.3) | 113  (11. 8) |
| 3. Li X | 54,30 | 23 (53,5) | 20(46.5) | 5 (11,6) | 38 (88,4) | 0 |
| 4. Wu M | 60.8 | 2150(75.54) | 696(24.46) | 44(1.55) | 2444  (85. 88) | 358  (12.58) |
| 5. Li Z | 61.7 | 997 (69.6) | 435 (30.4) | 406(28.4) | 942 (65.7) | - |
| 6. Zhan Z | 64.3 | - | - | - | - | 27 (9.4) |
| 7. Ji K  **Intestinal type** | 60.71 | 341 (79.1) | 90 (20.9) | 96 (22.3) | 313  (72.6) | 22 (5.1) |
| **Diffuse type** | 56.19 | 125 (57.6) | 92 (42.4) | 25 (11.5) | 182  (83.8) | 10 (4.6) |
| 8. Zhang XQ | 70.44 | 382 (77.6) | 110 (22.4) | 175 (35.6) | 317 (64.4) | 0 |
| 9. Kuwayama N | 67 | 1185 (70.2) | 502 (29.8) | 1171 (69.4) | 416  (24.7) | 100 (5.9) |
| 10. Chung H | 60 | 2569 (63.8) | 1456 (36.2) | 2709  (67.4) | 1304 (32.4) | 1. (0.2) |

Supplementary Table S2.2 summarizes clinical heterogeneity across studies, including differences in age distribution, sex ratio, and tumor stage, which may contribute to variability in model performance.

**
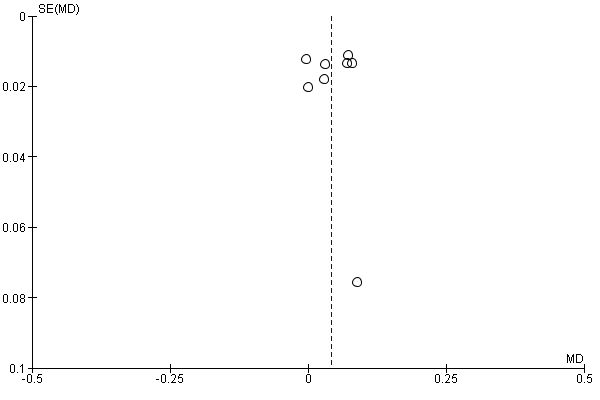
**

**Supplementary Figure S3.1- Funnel plot AI vs not AI**

**
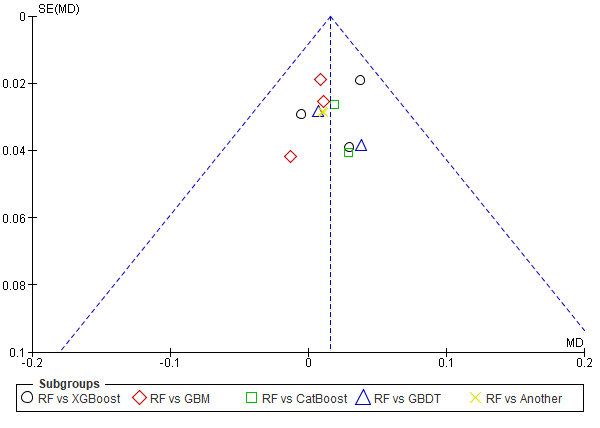
**

**Supplementary Figure S4.1- Funnel plot «bagging» vs «boosting» algorithms**


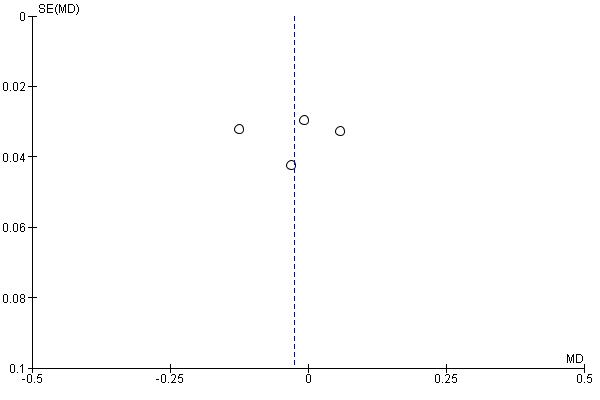


**Supplementary Figure S5.1 – Funnel plot SVM vs RF**


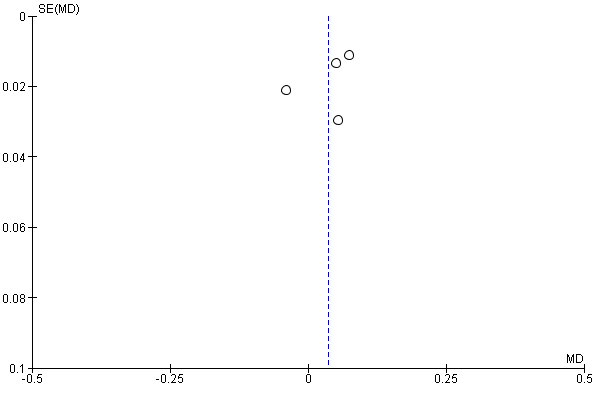


**Supplementary Figure S6.1.1–Funnel plot RF vs. Neural network algorithms**


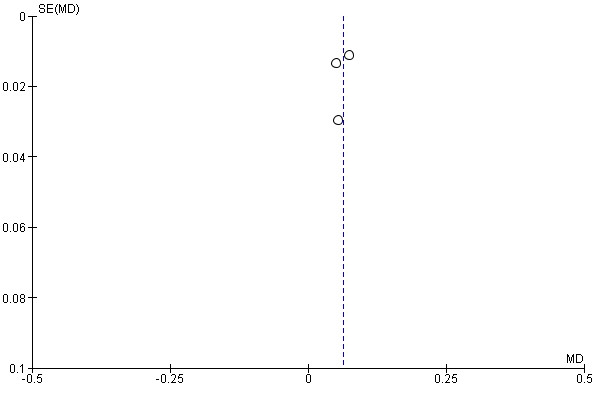


**Supplementary Figure S6.2.1–Funnel plot RF vs. Neural network algorithms (without Kuwayama N study)**


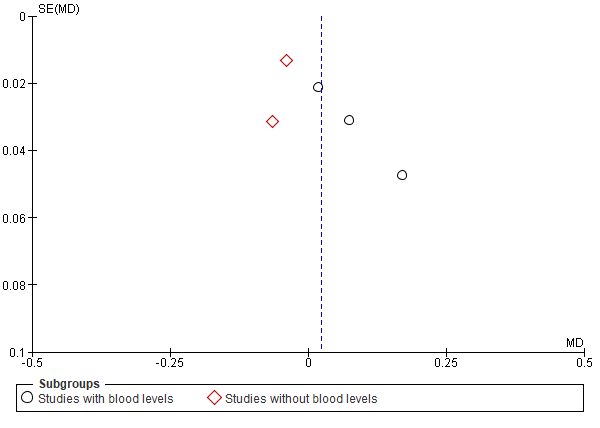


**Supplementary Figure S7.1 - Funnel plot RF vs LR**
